# Autistic psychiatrists’ experiences of recognising themselves and others as autistic: a qualitative study

**DOI:** 10.1192/bjo.2024.756

**Published:** 2024-10-30

**Authors:** Mary Doherty, Nick Chown, Nicola Martin, Sebastian C. K. Shaw

**Affiliations:** University College Dublin, Dublin, Ireland; London South Bank University, London, UK; and Brighton and Sussex Medical School, Hove, UK; London South Bank University, London, UK; Brighton and Sussex Medical School, Hove, UK

**Keywords:** Autistic, autism, neurodevelopmental, psychiatrists, qualitative

## Abstract

**Background:**

Diagnosis of autism falls under the remit of psychiatry. Recognition that psychiatrists could be autistic is recent. Psychiatrists are the second largest specialty group in Autistic Doctors International, a peer support group for autistic doctors.

**Aims:**

To explore the experiences of autistic psychiatrists in relation to recognising themselves and others as autistic.

**Method:**

This was a qualitative study using loosely structured interviews and an interpretive phenomenological analysis.

**Results:**

Eight autistic senior psychiatrists based in the UK participated. One had a childhood diagnosis, two had been diagnosed in adulthood and the remainder self-identified as autistic as adults. Recognition of autism followed diagnosis of their children or encounters with autistic patients. Barriers to self-recognition included lack of autism training, the deficit-based diagnostic criteria and stereotypical views of autism. Recognising that they were autistic led to the realisation that many colleagues were also likely to be autistic, particularly in neurodevelopmental psychiatry. All participants reported the ability to quickly recognise autistic patients and to develop a good rapport easily, once they were aware of their own autistic identity. Difficulties recognising patients as autistic occurred before self-recognition when they shared autistic characteristics and experiences. ‘If we don't recognise ourselves as autistic how on earth can we diagnose patients accurately?’

**Conclusions:**

Autistic psychiatrists face multiple barriers to recognising that they are autistic. Lack of self-recognition may impede diagnostic accuracy with autistic patients. Self-recognition and disclosure by autistic psychiatrists may be facilitated by reframing the traditional deficit-based view of autism towards a neurodiversity-affirmative approach, with consequent benefits for autistic patients.

Autism recognition is rising, particularly among the ‘lost generation’ of autistic adults who were not picked up in childhood and are now presenting for diagnostic assessment.^1^ Autism diagnosis usually falls under the remit of psychiatry. Although debate is ongoing as to whether this should be within specialist autism services or whether autism should be the business of every psychiatrist, there is little doubt that psychiatry is to the forefront in both setting the diagnostic criteria and assessing autistic people.^2^ The question of how to configure services to meet this demand is a topical debate in neurodevelopmental psychiatric discourse and healthcare service planning in the UK and beyond.^3,4^ Autism is commonly missed or misdiagnosed.^5^ Recent research spotlights the high prevalence of previously unrecognised autism among people presenting to mental health services across all settings, including in-patient and out-patient services and crisis presentations to emergency services.^6–9^ Recognising underlying autism in such cases may provide the key to positive outcomes, yet healthcare providers report lack of knowledge and confidence in doing so.^10–11^

It has more recently been recognised that psychiatrists themselves could be autistic. A study exploring the knowledge, attitudes and experiences of psychiatrists in the UK in relation to autism recruited 172 participants, of whom two identified as autistic.^12^ The first publication on autistic psychiatrists appeared in 2022, calling for their inclusion in the Royal College of Psychiatrists Equality Action Plan.^13^ Little is known about the experiences, perspectives and insights of autistic psychiatrists. A study of autistic doctors across all specialties found psychiatrists to be the second largest specialty group within the peer support group Autistic Doctors International (ADI).^14^ It is not known whether this reflects a wider prevalence of autism among psychiatrists. Most autistic adults may not realise they are autistic.^15^ However, we do not know whether this is also true for psychiatrists. Published accounts from autistic psychiatrists are rare, and it may be that most autistic psychiatrists who are self-aware do not disclose.^14,16,17^

Autism is currently diagnosed based on externally observed differences from a perceived norm.^18^ Embodying such differences may in itself inhibit the recognition that one is actually different from the majority. Alternatively, autistic strengths such as enhanced pattern recognition may aid self-recognition. There has been no research exploring the impact on autistic psychiatrists of their awareness of their own autistic nature, either for their own benefit or that of their patients. Considering the core role of psychiatrists in the recognition and diagnosis of autism, this is a topic warranting investigation. This project is part of a wider doctoral study exploring the experiences of autistic psychiatrists. For this paper, our overarching research question was: ‘what are the experiences of autistic psychiatrists in relation to recognising autism?’

## Researcher positionality

Our team spans a range of positionalities from total outsider to partial insider. We have autistic and non-autistic representation. We also include two of the core leads of ADI, a peer support and advocacy group for autistic medical doctors. Although our team does not include psychiatrists, two of our authors are medical doctors, both of whom have past experience of working in psychiatric settings as junior doctors. We acknowledge that there are differences of opinion regarding medical, social and neurodiversity models within the autism clinical, patient and carer communities. Having found an association in our previous research between viewing autism as a disorder and prior suicide attempts,^14^ we are unequivocal in approaching this topic from a neurodiversity-affirmative standpoint.

## Method

### Methodology

This qualitative study draws on an interpretive phenomenological methodology. Phenomenology is the study of human experience and, in particular, the elements of lived experience which are meaningful to individuals.^19^ Interpretive phenomenology acknowledges and embraces the researcher's insider perspective, prior knowledge and potential bias in conducting the research and uses this to access experiences and data from participants which might not otherwise be obtained.^20^ Interpretive phenomenological analysis seeks to explore the experiences of a relatively small, homogeneous group of participants who undergo a shared experience or who experience a shared phenomenon, while retaining an idiographic focus on individual participants.^21^

### Data collection

Purposive sampling was used. Participants were invited to partake via a message posted to the ‘ADI Psych’ WhatsApp group, a psychiatry-specific subgroup of ADI. Eligibility criteria were being a psychiatrist, not in training, and identifying or formally diagnosed as autistic. A topic guide was developed, and considerations around research with known participants were explored.

Written, informed consent was received from all participants before scheduling and reconfirmed at the start of the interview. Data were collected using loosely structured online interviews via the Zoom platform, which were video recorded and transcribed verbatim. Interviews lasted a mean of 88 min (range: 66–143 min). See Supplementary material available at https://doi.org/10.1192/bjo.2024.756 for the interview topic guide.

### Data analysis

This followed the framework described by Smith and Nizza.^22^ Recordings and transcripts were reviewed several times. Transcripts were annotated with exploratory notes, which were then used to develop a series of experiential statements. Clustering of experiential statements and construction of a table of personal experiential themes (PET) led to an overall analytical framework for each participant, which aimed to capture the essence of the most important and meaningful aspects of that participant's story. All groups of PETs were then reviewed together, looking for common threads across the participants. Each inductively constructed table was highly personal to each participant, but similarities in the overall experiences were evident on first review. A deeper exploration looking for connections, similarities and discrepancies was conducted, resulting in the development of group experiential themes (GETs). The GETs highlighted areas of similarity between participants but also areas of divergence. An iterative process involving multiple drafts and revisions ultimately resulted in the data-set being divided into broad topic areas to be reported separately. Reflexive participant collaboration was used.^23^ Participants were offered the opportunity to review transcripts and to confirm the omission of any potentially identifying data. Following data analysis, they were offered the opportunity to read the draft paper and confirm the accuracy of their individual representation and comment on the analysis and interpretation. This process resulted in the removal of a single phrase from one participant quote. The analysis presented here interrogates the experiences of autistic psychiatrists specifically in relation to recognising autism.

### Ethics

The authors assert that all procedures contributing to this work comply with the ethical standards of the relevant national and institutional committees on human experimentation and with the Helsinki Declaration of 1975, as revised in 2013. All procedures involving human subjects/patients were approved by London South Bank University Research Ethics Committee; ETH2122-0128.

## Results

### Demographics

Eight participants were recruited: six consultant psychiatrists and two senior associate specialists. All were based in the UK. Seven were currently employed, and one was retired. Four were child and adolescent psychiatrists; the others were old age, liaison and general adult psychiatrists. Two had formal autism diagnoses received in adulthood. One had been diagnosed with pervasive developmental disorder – not otherwise specified (PDD-NOS) in childhood and identified as autistic as an adult. The remaining five self-identified as autistic as adults. Pseudonyms have been used and identifying details changed or omitted to preserve anonymity. See Table 1 for details of participant demographics.

**Table 1:** Participant demographics

Name	Alistair	Fatima	Joanne	Lisa	Michelle	Paula	Sandra	Trevor
Age, years	75–80	40–45	40–45	35–40	45–50	40–45	35–40	50–55
Gender	Male	Female	Female	Female	Female	Female	Female	Male
Ethnicity	White/other	Indian	White British	White/other	White British	White/other	White British	White/other
Employment	Retired	Full-time	Full-time	Full-time	Part-time	Part-time	Full-time	Full-time
Experience in psychiatry, years	>40	10–15	10–15	10–15	20–25	15–20	10–15	15–20

The analysis resulted in three GETs related to the recognition of autism.

### Theme 1: recognising oneself as autistic

The realisation that they were autistic dawned in different ways for participants. For some, it was following the identification or diagnosis of their children. Others experienced a gradual awareness during medical training. The shock of encountering a specific patient who reminded them of themselves heralded recognition for two participants. One participant always knew, following a childhood diagnosis.

Michelle recognised shared experiences with her son, which led her to wonder if she was also autistic.

‘*All sorts of little things that I read up about were ringing bells and I was seeing that I had sort of milder versions of what I was seeing in my son.’*

In preparation for her son's assessment, Michelle had prepared a voluminous account of his potentially autistic features. She was asked during the assessment if she had considered that she might also be autistic. Michelle had indeed considered this but had discounted it.

‘*I said “oh I think I know why you might think that”, thinking the report was very long … “but I think I'm weird for other reasons”.’*

It was some years before Michelle realised that she was indeed autistic too, and she expressed deep gratitude that the seed was planted by her son's psychiatrist.

‘*I will be eternally grateful to her for just that one line, just that one line was all it took.’*

Parenting of autistic children leading to self-recognition was a common experience for several participants. Joanne initially recognised that her children were autistic then ‘sort of very gradually built towards me too.’ She had not sought formal diagnosis for herself or her children because she was certain about their autistic identity without a need for external validation.

*‘I know we all are [autistic]; I don't really feel like I need anyone else to tell me that … I do it at work so I know exactly what to look for and I tick well over the amount that needs to be ticked to be a yes.’*

Fatima described the poignant realisation of her own autistic nature, having long assumed her son had inherited autism from his father.

‘*He is quite typically … I mean the way the textbooks describe it, how autism is in men so it's not difficult to see that in him … I never entertained the possibility that it could be me, but 2 years ago … I realised that … it's always been me.’*

Other participants came to the realisation following encounters with autistic patients. Sandra was diagnosed as autistic 3 years ago, having self-identified many years previously. She was surprised to receive a simultaneous diagnosis of attention-deficit hyperactivity disorder (ADHD) despite her description of ‘having this magpie sort of brain that got jammed’. She initially recognised herself as potentially autistic during a medical school lecture on autism and then again during her psychiatric training.

*‘I very much enjoyed my CAMHS placement [child and adolescent mental health], and I really felt most at home with the seven-year-old autistic ADHD children … I thought “these are my people”.’*

Trevor described a complicated and unconventional family background. Throughout his life, his unusual characteristics had been attributed to repeated family relocation and upheaval. However, he recognised that autism was the more likely explanation.

‘*It makes a lot of sense of bizarre things [which] have happened in my life and odd things about how I am.’*

Trevor did not have a formal autism diagnosis and was confident enough in his own autistic identity to not need external validation. Although he had not disclosed formally, he was open with both colleagues and patients.

*‘I … make a bit of a joke of it at times actually, because I think it's an open secret among some of my colleagues and indeed some of my patients that I'm probably autistic.’*

Several participants reported having been recognised as autistic by patients. Trevor experienced validation of a community-confirmed autistic identity by several autistic colleagues and patients.

‘*I started bumping into people, colleagues with autism and patients with autism who just said … “you're one of us, aren't you”?’*

Lisa was unique among the study participants in that she received a childhood diagnosis (aged 5 or 6) of PDD-NOS, which was subsumed into the category of ‘autism spectrum disorder’ in the DSM-5 in 2013. Lisa was certain her childhood self would be diagnosed as autistic today.

‘*No doubt … well they have removed the PNDs [pervasive developmental disorders] from the diagnostic criteria anyway, but I think you know the way we kind of think about it in a slightly less rigid way and a bit more used to female presentations and not lumping [autism] in with learning disability.’*

Lisa displayed a deeply positive self-identity and a self-assurance that set her apart from other participants. She was aware of her somewhat unique position among her neurodivergent colleagues as she observed the struggles of late-identified adults.

‘*I'm the only person [in the staff neurodiversity network] that actually had a childhood diagnosis … and you know they have all this … I don't know, internalised ableism or whatever … growing up and having no clue why everything is a struggle … ’*

Paula recalled a confusing encounter with a teenage patient who reminded her of herself but who was suspected to be autistic. Paula subsequently recognised the possibility that she was autistic during a webinar.

‘ *… about managing autistic people … in mental health crisis, and I thought … oh.’*

Afterwards, Paula tentatively questioned a friend, a clinical psychologist who had known her ‘quite closely throughout the trajectory of [her] whole life’, having been classmates throughout primary and secondary school, and housemates at university.

‘*Do you think there's any chance or am I being a total hypochondriac here … could I have like the girl's version of ASD?’*

Paula was stunned and felt somewhat humiliated by her friend's reply.

‘*She went “how's that taken you so long to realise” and she's like “I thought you knew already” … I was like … “well, no”.’*

The revelation was a challenge to Paula's self-image as insightful and self-aware, and she wondered who else might have known.

‘*When she said that I felt a bit like oh gosh … so she's known this behind my back and not said anything and then I felt a bit like humiliated … maybe other people would know … [I] felt a bit thick being a psychiatrist who's had seven years of therapy who would diagnose and recognise autism in other people … For me not to realise that until like 40 was a bit like … whoa, that's ridiculous.’*

For Alistair, the assessment and diagnosis of a junior doctor with whom he strongly identified was a transformative experience which forced him to confront his own autistic nature, yet he remained somewhat conflicted and struggled with his own identity as an autistic doctor. He was reluctant to give an autism diagnosis, as that would require an acknowledgement that he was also autistic.

‘*He didn't come across as floridly autistic … but there were anecdotes, I thought … I've been there, done that … ’*

*‘I felt terribly uncomfortable; I did a lot of telephoning and speaking to other people who had worked with him and saw him in [multiple] settings and decided yes, he did have autism … and … what did that say about me?’*

Coming to terms with potentially being autistic took time and was intensely uncomfortable for Alistair.

‘*It took me about three months to get comfortable with the idea that maybe I did have autism, maybe.’*

The tension between recognising himself as autistic and the idea of autism as disorder was a recurrent consideration for Alistair as he pondered

*‘ … how do you set your stance of normal?’*

### Theme 2: recognising autistic colleagues

There was an undeniable interplay between self-recognition as autistic and the subsequent realisation that colleagues may be autistic too. Although this manifested in a variety of ways for different participants, this temporal relationship remained central. Alistair spent his career in the field of intellectual disability, so the realisation that ‘there are autists wandering free in the world’ was a shock. It led him to recognise a shared identity with numerous colleagues, especially in neurodevelopmental services.

‘*I mean a number of colleagues, I look at them I think yes … I've learned their rather odd behaviour … but that's because I've got into the field of psychiatrists who are interested in autism and there's a huge degree of self-selection.’*

Describing a close colleague who was exploring an autistic identity for themselves, Alistair alluded to the inhibition felt by potentially autistic psychiatrists, the use of euphemism as a distancing tactic and the reluctance to openly acknowledge what remains an intensely stigmatised label.

‘*a roughly similar path to mine and he sort of talks about his Aspie traits but not saying he's autistic, not saying it, just that sort of passing reference on a couple of occasions.’*

Alistair had not shared his own autistic identity, even with this colleague, whom he described as a friend. He recognised the increasing phenomenon of colleagues coming to explore the possibility, and the common pathway which each one seemed to tread in isolation.

‘*I think a lot of them are sort of running down the same road I've been going down of gradual realisation and this is because looking around psychiatrists there are those that are clearly autistic and … usually pretty involved in neurodevelopmental disorder.’*

Other participants recognised consultant colleagues as likely to be autistic. These quotes are intentionally separated from their participants to protect against possible triangulation of their identities.

‘*some of my colleagues in the team, whether or not they've disclosed it and whether or not they're in touch with it, are also on the spectrum.’*

‘*From observation I would say it is highly likely that a colleague who has autistic siblings is autistic … and they regard themself as the “normal” one … which I find a bit funny.’*

*‘a high probability at least two other consultants in my immediate vicinity are autistic … both of whom are noticeably eccentric … ’*

‘ *… my clinical director is [probably] autistic.’*

Sandra was unique among the participants in that she worked with ‘quite a few neurodivergent people’ including openly autistic colleagues, although she had not herself disclosed.

‘*a lot of people who are attracted into the fields that I work in are probably neurodivergent in some way.’*

She did not find this surprising.

‘*Looking back over my … years of doctoring … I think it actually very much suits a neurodivergent brain.’*

She was unsure if she is recognised as autistic by others.

‘*I think because there's so much of it about people think well, I'm no different to my colleagues.’*

Participants were keenly attuned to their colleagues’ potentially autistic features, yet they also recognised obvious differences between themselves and non-autistic psychiatrists. This suggests a balanced perspective borne out of increased awareness rather than participants having a confirmation bias towards autism, even in relation to colleagues within neurodevelopmental services.

‘*there are … well, a number of people involved in autism who really have no obvious [autistic traits] … are socially skilled, communicate well, have all the talents that I wished I had.’* (Alistair)

### Theme 3: Not recognising autistic patients

All participants reported the ability to quickly recognise autistic patients, including those missed by colleagues, and to develop good rapport easily, once they were aware of their own autistic identity. However, several recounted challenges earlier in their journey. Alistair reflected on a long career in academic clinical practice and involvement in development of diagnostic tools still used today. His lack of awareness of his autistic nature had potential implications for the validity of the instruments developed, as many of the items apparently lacked specificity.

‘*I remember arguing about rating scales and things … how would they answer that question, this question; or this question seemed quite inappropriate because you know someone like myself could say “yes that's me” therefore it wasn't really a good one for picking up [autism].’*

In clinical practice, Alistair's early lack of self-awareness made it more difficult to recognise autistic patients.

‘ *… that really came home with the … houseman; well, well, it was just he was so like me (laughing)’*.

Paula described a similarly impactful patient encounter. She experienced deep empathy and resonance with a teenage patient who reminded her of her younger self. However, Paula experienced the cognitive dissonance arising from seeing her patient as autistic but not (yet) herself.

‘*I really felt for her … that's how my mind works as well … but I could see I was suspicious this girl was on the autistic spectrum, but I thought she's like me but … I'm not on the autistic spectrum.’*

Paula was introduced to the concepts of masking, social scripting and the female presentation of autism during the aforementioned webinar. Her initial reaction to the description of female autistic features was ‘that's normal’.

‘*I've never heard of masking before but they were talking about women masking and doing things like inventing social rules to survive social situations … I thought well that's normal I would do that … that's just being clever isn't it, being clever and strategic, why is that autistic?’*

Although Paula did later recognise that ‘obviously my benchmark for what's normal is me’, at the time her reaction was ‘well we all do that don't we, meaning I do that so therefore obviously everybody else does.’ She felt confused. Yet, she remembered her young patient with a dawning recognition of the reason for the resonance.

Dominant male stereotypes and lack of knowledge of female autism or co-occurring ADHD led to a surprising outcome for Sandra, despite self-identifying as autistic herself at the time.

‘*It was very much … this sense of it being a predominantly male thing … certainly there weren't at that point very many female autistic people to … identify with … I suggested that someone I knew, a female, be assessed for ADHD … they … were told that they were likely autistic as well … that made me … really think about female autism in a very different way … it made me think I've been perhaps missing patients.’*

Michelle took a wider perspective and shared the experience of many of her autistic colleagues along with her own.

‘*The autistic psychiatrists’ perspective – we don't recognise ourselves from training. That's been the overriding feeling for me and for many other autistic psychiatrists I've spoken to.’*

Limited and stereotypical views of autism were related to a lack of exposure to the heterogeneity among autistic people during medical school and postgraduate training, and consequently Michelle could not conceive of a potential application to herself.

‘*We never thought we could be autistic; it never even occurred to us because training on autism has been so small as a medical student and then not a huge amount unless you're in learning disability … as a psychiatrist you know the diagnostic criteria and you would recognise … stereotypical cases of autism no problem but nothing beyond that and … I mean I've never thought about really what those criteria meant before’*.

In their prior education and training, participants reported a lack of recognition that people who were outwardly successful could be autistic. This led to difficulty recognising themselves, and subsequently patients, as autistic.

‘*People in professional jobs with relationships and children … are all getting missed … our yardstick as doctors is skewed … and so the overriding problem is that if we can't recognise ourselves as autistic how do we recognise other people that are autistic.’* (Michelle)

Patient experiences which seemed familiar to a clinician risked being disregarded, leading to inaccurate assessments when a clinician was actually autistic but unaware and erroneously assuming they were not.

‘*So if we're saying when we see a patient “well I do that” even if we're saying it in our heads … we're saying that because we think we're not autistic … well actually we don't even know that we're not autistic and a lot of us have turned out to be autistic … so if we don't recognise ourselves how on earth can we diagnose the patients accurately?’(Michelle)*.

The benefits of realising they are autistic were recognised by all participants, potentially being ‘life-changing for some patients’ (Joanne). Paula noticed the personal impact of a neurodiversity-affirmative framing of autism and speculated as to the potential impact in terms of mental health.

‘ *… because I've noticed in myself that it's boosted my self-esteem … I think it would help others as well … that will have a benefit on … how you feel about yourself … and could be then preventative of any mental illness.’*

In Fatima's case, recognising that she was autistic, that she had been different rather than abnormal, had profound implications for her patients.

‘*I started seeing the same things in young people, that's when it occurred to me … there's nothing wrong with them because nothing was wrong with me and yet they have behaved exactly the same.’*

Michele also described the positive impact.

‘*it's still an absolute revelation to discover you're just a good autistic not a failed non-autistic so the mental health implications are still huge.’*

She went on to describe the guilt that follows the recognition that some patients may have missed out on these benefits.

‘*So as a psychiatrist we've all got this guilt that we've missed all these people … whichever branch of psychiatry you're in you have missed people … you have thought in your head at least if you even thought of autism, a lot of the time you wouldn't even thought of it, but if you have you kind of rattled through the very basic criteria and gone no, no, no … but in retrospect we all realise how many people we've missed.’*

Joanne described the professional satisfaction of using her knowledge of autism to benefit her patients and related it back to her own journey to autistic identity.

‘*It's really satisfying … and if I had not had the experience I had with the kids, I wouldn't have picked that up so … when you think about the whole journey it sort of all comes together.’*

## Discussion

This qualitative study explored the experiences of autistic psychiatrists in relation to autism recognition. Their potentially enhanced ability to recognise autism once they had self-identified or been diagnosed may offer benefits to both patients and colleagues. Their individual journeys to autistic identity, recognising themselves as autistic, led to the subsequent recognition that many colleagues in psychiatry – particularly in neurodevelopmental psychiatry – were also potentially autistic. Such psychiatrists may be disadvantaged on a personal level by not recognising themselves as autistic, but there are also implications for patient care. Perceived similarities between patient and psychiatrist may act as a confounder in clinical encounters.^24^ Our data suggest that psychiatrists not recognising themselves as autistic potentially risks under-recognition of autistic people attending for assessment or utilising mental health services.

Autistic doctors are a hidden minority in the healthcare workforce.^25^ Psychiatrists may fail to recognise themselves as autistic for many reasons. Undergraduate and postgraduate teaching on autism usually relates to deficit-based, stereotypical presentations, which are often conflated with learning disability.^26^ There is a lack of lived experience co-delivery of autism education and training, which could help to shift attitudes and understanding towards a more neurodiversity-affirmative perspective. Autism is an intensely stigmatised diagnostic label, with negative assumptions around empathy and theory of mind.^27^ Changes in the conceptualisation of autism and indeed the diagnostic criteria have had positive impacts, but myths, misconceptions and stereotypes prevail.^28^ The diagnostic criteria for autism remain deficit focused and do not appear to include the possibility of an autistic person thriving.^18^ Therefore, successful psychiatrists may fail to see themselves reflected in these criteria. Another possibility is not recognising autism alongside ADHD. Until the publication of the DSM-5 in 2013, autism and ADHD were deemed to be mutually exclusive.^29^ Although it is increasingly recognised that co-occurrence with other neurodevelopmental conditions is the norm rather than the exception, the traditional separation may have a persisting impact.

Resource constraints and gatekeeping in autism services may perpetuate difficulties in recognising autistic people not in obvious difficulties.^30,31^ Service capacity limitations commonly mean that those with co-occurring mental illness or significant challenges are prioritised for diagnostic assessment.^31,32^ Autistic people who are thriving – those who are in employment and perhaps have families, and are without obvious major challenges – are deprioritised and may not meet the perceived threshold for clinical assessment.^33,34^ This reinforces the association between being autistic and being in difficulty, which makes it harder to recognise oneself when one is successful and well.^35^

The recognition of one's autistic nature is commonly perceived as life-changing in a positive way for late diagnosed autistic adults.^36^ It is an opportunity to reframe one's perspective and realise that rather than being a failed version of a neurotypical person struggling to exist in a neurotypical world, one is a perfectly good autistic person – different rather than disordered or defective.^37^ For psychiatrists caring for patients experiencing mental illness, recognition of underlying autism brings the possibility of offering reasonable adjustments and a reframing of personal identity in line with a neurodiversity perspective, which may improve mental health outcomes.^10,38^

Neurodivergent and autistic traits are common and are increasingly recognised as valuable in medicine.^17^ Considering personal and patient-centred benefits of self-awareness and the apparent concentration of potentially autistic psychiatrists in neurodevelopmental services suggested by our data, it may be that all psychiatrists, and particularly those in relevant services, may benefit from considering their own autistic traits and their own position along the spectrum of neurodivergence. Self-reflection is already a core element of training and practice for many psychiatrists, although adding consideration of neurodivergence may seem novel.^39^

Following self-recognition, the question of disclosure is a significant consideration. The experiences and perspectives of autistic psychiatrists in relation to disclosure, including reasons for non-disclosure, form another part of this wider doctoral project and will be reported in depth elsewhere. Our findings in this paper suggest that not recognising one's own autistic nature may risk missing an autism diagnosis for patients presenting for autism assessment or mental healthcare. Recognition of one's own positionality is a private affair and in no way implies a need to disclose to anyone else. Personal and patient benefit is achieved on recognition, independent of disclosure, which may carry professional risk.^17,40^ However, potential implications of non-disclosure in psychiatric education and practice warrant consideration at a wider cultural level. Although it is important to avoid speculation in relation to individual colleagues, it should not be assumed that a psychiatrist not disclosing an autism diagnosis is not autistic. Making this assumption risks imposing a similar confounder to a lack of self-recognition, in terms of recognising autistic patients, as psychiatrists risk missing patients who present with similar mannerisms and communication styles to colleagues who are autistic but not openly so. The range of autistic presentation is vast; if the wide variability is not recognised, autistic patients may continue to be missed.

The Royal College of Psychiatrists is actively working to support autistic psychiatrists in response to calls to recognise their existence and meet their needs.^16,17^ Future projects might explore the support needs of potentially autistic psychiatrists, particularly those who are tentatively exploring personal relevance. This is currently a lonely path to tread. Challenges are exacerbated when colleagues or professionals in support roles question the possibility that doctors can be autistic.^40^ Peer support groups including ADI are open to those confident in their autistic identity, whether formally diagnosed or not, but there is a dearth of resources for those wishing to explore the possibility.

### Strengths and limitations

The partial insider status of the research team is a particular strength of this study, as experiences of autistic psychiatrists, an unrecognised and stigmatised group of doctors, may have been inaccessible to other researchers. As this was a qualitative project, we do not claim generalisability, but the analysis was a deep and nuanced exploration of our participants’ experiences. Trainee psychiatrists’ voices were not included; this was a limitation, although a deliberate choice. Interpretive phenomenology seeks to explore the experiences of a small homogenous group, but intersectionality was represented in terms of gender, ethnicity and sexuality among participants.

We acknowledge that a minority of our participants had an official autism diagnosis. The reasons for seeking or not seeking a formal autism diagnosis will be reported elsewhere. The accuracy of self-identification in psychiatry is a contentious area, particularly in relation to autism.^41,42^ However, our participants were psychiatrists whose clinical role involves autism diagnosis. Membership of ADI Psych involves scrutiny by other psychiatrists, and acceptance within the group offers community confirmation when the group consists of psychiatrists who regularly diagnose autism.

### Clinical implications

Autistic psychiatrists face multiple barriers to self-recognition as autistic. Their insights have the potential to reframe our understanding of autism and improve healthcare outcomes for the autistic community, including services and outcomes for autistic people in psychiatry, whether as patients or service providers.^10,43^

The need for autism training across all healthcare settings and professional groups is undisputed.^44^ The experiences, perspectives and insights of autistic psychiatrists have potential to inform such practice, yet ableism in medicine and the stigma associated with an autism diagnosis may impede recognition; this may have negative implications for patients. Increasing visibility of traditionally under-recognised groups of autistic people, particularly women, those from ethnic minority backgrounds and multiply neurodivergent individuals, may also assist self-recognition by autistic psychiatrists. The Autistic SPACE framework, although developed to meet the needs of autistic people in healthcare settings, may also assist a potentially autistic psychiatrist in identifying their own positionality and autism specific needs.^45^ Self-recognition and disclosure by autistic psychiatrists may be facilitated by reframing the traditional deficit-based view of autism towards a neurodiversity-affirmative approach, with consequent benefits for autistic patients.

## Supporting information

Doherty et al. supplementary material Doherty et al. supplementary material

## Data Availability

The data that support the findings of this study are not available to share. Our ethical approvals do not support data-sharing, and our participants did not consent to such data-sharing. Our data are highly sensitive, and the personal accounts are potentially identifiable. We have included anonymised quotes throughout our results section to evidence the analysis and support our interpretation. Ethical queries can be addressed toethics@lsbu.ac.uk
