# Supplementary material for: Autistic psychiatrists’ experiences of recognising themselves and others as autistic: a qualitative study
Source: BJPsych Open. 2024 Oct 30;10(6):e183. doi: 10.1192/bjo.2024.756 (PMC11698211; doi:10.1192/bjo.2024.756)
Supplement: Doherty et al. supplementary material [file S2056472424007567sup001.docx]

| **Broad topic area** |  | **Possible example questions** |
| --- | --- | --- |
| Training, professional role |  | Tell me about yourself |
| Diagnostic status |  | Have you sought a formal diagnosis? Why or why not? |
| Route to self-identity or diagnosis |  | How did you come to recognize you were autistic? |
| Disclosure status |  | Have you disclosed to anyone at work? |
| Disclosure experiences or barriers |  | How did you tell them / break the news?  What were you thinking at the time?  How did you feel after you told them? |
| Impact on career |  | How has disclosure impacted your career? |
| Self-perception as autistic psychiatrist |  | How do you come to conceptualize your identity as an autistic psychiatrist? |
| Perceptions of autistic patients by psychiatrists |  | What is it like hearing fellow psychiatrists discuss autistic patients? |
| Treating autistic patients as an autistic psychiatrist |  | What is it like treating patients who are autistic? |
|  |  |  |
| Anything else |  | Is there anything I have missed or not covered? |
|  |  | Any other experiences you want to talk through? |
